# Collaborative Research With Community-Based Healthcare Providers: Co-Presented Learnings From Pilot Studies

**DOI:** 10.1093/geroni/igaf122.593

**Published:** 2025-12-31

**Authors:** Kali Thomas, Emily Gadbois

## Abstract

This symposium highlights five community-engaged research projects conducted by the Brown University Center for Long Term Care Quality and Innovation (Q&I). Each symposium will be co-led by a community partner and a Q&I Center researcher. In each presentation, we will describe the processes for project prioritization, collaborative outcome selection, data acquisition, co-interpretation of key findings, and recruitment and consent decisions. We will also describe site- and population-specific barriers to conducting pilot studies. Community partners include: Meridian Senior Living, which owns and operates over 50 assisted living communities (ALCs) across 21 states; Bluestone Physician Services, which provides chronic disease management to over 15,000 patients at approximately 575 ALCs in three states (Florida, Minnesota, and Wisconsin); CareLink, a Rhode Island-based healthcare network providing high-quality, evidence-based care for over 5,000 older adults each year; and PACE RI, a health plan which has provided care to over 2,000 nursing home eligible, community-dwelling adults in RI, with the goal of maintaining independence. We will transparently discuss our failures and successes and share tools that may be helpful to participants conducting community-engaged research.

